# Recombinant influenza H9N2 virus with a substitution of H3 hemagglutinin transmembrane domain showed enhanced immunogenicity in mice and chicken

**DOI:** 10.1038/s41598-017-18054-x

**Published:** 2017-12-20

**Authors:** Yun Zhang, Ying Wei, Kang Liu, Mengjiao Huang, Ran Li, Yang Wang, Qiliang Liu, Jing Zheng, Chunyi Xue, Yongchang Cao

**Affiliations:** State Key Laboratory of Biocontrol, College of Life Sciences, Sun Yat-sen University, Guangzhou, 510006 P. R. China

## Abstract

In recent years, avian influenza virus H9N2 undergoing antigenic drift represents a threat to poultry farming as well as public health. Current vaccines are restricted to inactivated vaccine strains and their related variants. In this study, a recombinant H9N2 (H9N2-TM) strain with a replaced H3 hemagglutinin (HA) transmembrane (TM) domain was generated. Virus assembly and viral protein composition were not affected by the transmembrane domain replacement. Further, the recombinant TM-replaced H9N2-TM virus could provide better inter-clade protection in both mice and chickens against H9N2, suggesting that the H3-TM-replacement could be considered as a strategy to develop efficient subtype-specific H9N2 influenza vaccines.

## Introduction

H9N2 avian influenza virus was first isolated in turkeys in 1966^1^. Since then, it became prevalent in poultry farming worldwide, resulting in egg production reduction and high mortality when co-infected with other pathogens^2,3^. Also, it could cross host-species barrier and cause human infections as reported in China^4,5^. Though it is not highly pathogenic as H5N1, researches revealed that it could re-assort with multiple other influenza subtypes and thus be “gene donor” for H5N1 and H7N9 viruses^6–8^. Therefore, control of the H9N2 influenza virus is of great concern.

Vaccination utilizing vaccine strains and their relevant variants is the main strategy to control H9N2 pandemics in the poultry industry of China. However, the vaccine strains and their antigenic variants undergoing antigenic drift were responsible for the outbreak of H9N2 in the poultry farming of China during 2010–2013^9^. Lack of cross-immune protection in the existing vaccines becomes a severe problem of effective protection against the virus. Thus, a broad-spectrum vaccine which can provide cross-protection against different antigenic H9N2 variants is in urgent need.

The genome of avian influenza virus contains a single-stranded, negative-sense segmented RNA that encodes 12 proteins including hemagglutinin (HA)^10^. Hemagglutinin (HA) is recognized as the major surface antigen. It is critical for viral attachment and membrane fusion. These are key steps for virus’s entre into cells and critical to further process of virus infection^11,12^. Transmembrane (TM) domain of the HA protein serves as an anchor site and plays an important role in supporting viral fusion to the target membrane^13^. In acidic environment, substitution of the TM domain showed an abolishment of receptor binding and membrane fusion, leading to a failure of virus entry into the cells^14,15^. In addition, this domain was found to be important for biological characteristics of the influenza viruses, such as viral replication, virulence and pathogenicity^16–18^. Our previous research showed that substitutions of cysteines in the HA TM domain and a replacement with the H3-HA transmembrane (TM) domain could enhance heterosubtypic protection (hetero-protection) in mice^19^. And an inactivated recombinant H7N9 vaccine with the TM-replacement presented broadened protection with promoted HI titers in vaccinated animals using antiserum. Furthermore, the level of IFNγ was also increased, when inactivated H7N9 viruses were used as stimulant^20^. Therefore, the HA TM domain is considered to be a potential candidate site for vaccine development.

In this study, we generated a recombinant H9N2 wild type strain (H9N2-WT) and a recombinant H9N2 strain with a H3-TM domain replacement (H9N2-TM) utilizing reverse genetics system. The biological characteristics and immunogenicity between the two viruses were compared. Our results showed that the replacement of transmembrane (TM) domain did not affect the virus assembly and viral protein composition in the recombinant H9N2 viruses. However, the biological characteristics, such as virus growth, ratio of trimer, thermal stability, acidic resistance and fusion activity were altered, suggesting an important role of the TM domain in viral replication and pathogenicity. Furthermore, the TM-replaced H9N2-TM strain exhibited better protection in both mice and chicken when challenged against different phylogenetic H9N2 clades.

## Results

### Replacement of H3 HA TM domain did not affect the assembly and viral protein compositions of recombinant H9N2 viruses

**Figure 1:**
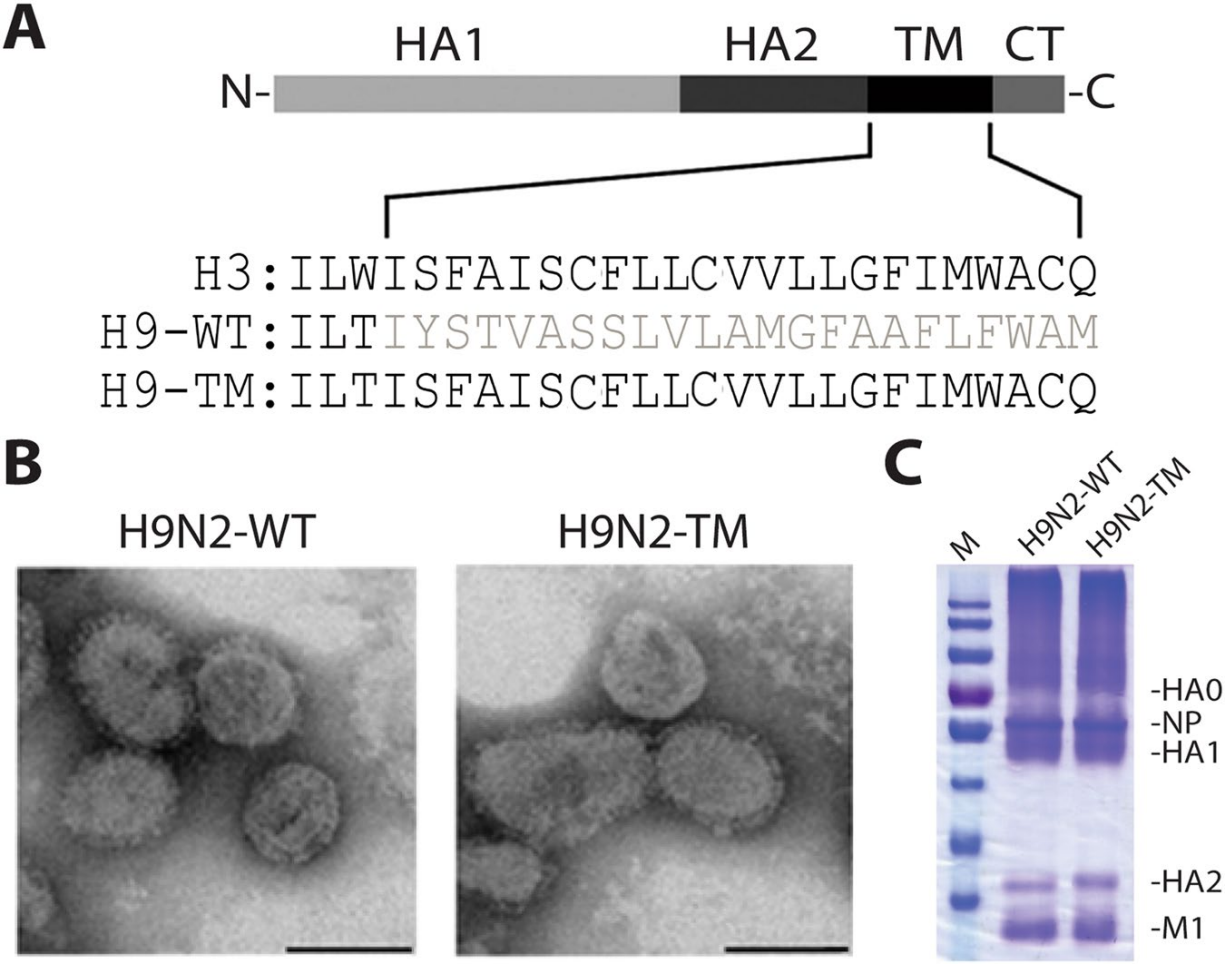
TM-replacement did not alter the assembly and viral protein compositions. (**A**) Structural schematics of influenza hemagglutinin (HA) proteins. The replaced transmembrane (TM) domain is marked grey. (**B**) Electron images of negatively stained purified H9N2-WT and rH9N2-TM virus particles. Scale bar is 100 nm. (**C**) SDS-PAGE of viral components of H9N2-WT and H9N2-TM.

To understand whether change of transmembrane (TM) domain can affect virus structure, we first observed the morphology of TM-replaced viruses rescued by reserve genetics. Applying electron microscope, the recombined TM-replaced virus (H9N2-TM) showed typical surface spikes as the recombined wildtype (H9N2-WT) (Fig. 1B), suggesting the replacement of the transmembrane (TM) domain did not change the surface structure of the virus. SDS-PAGE showed that the expression levels of HA0, HA1, HA2, NP, and M1 proteins were comparable in the two viruses (Fig. 1C). Full-length blot is presented in Supplementary Figure 1A. These results suggest that the replacement of H3 HA transmembrane (TM) domain does not affect the assembly and viral protein compositions of recombinant H9N2 viruses.

### TM-replaced virus showed reduced viral growth rate and better adaptation to chicken embryos

**Figure 2:**
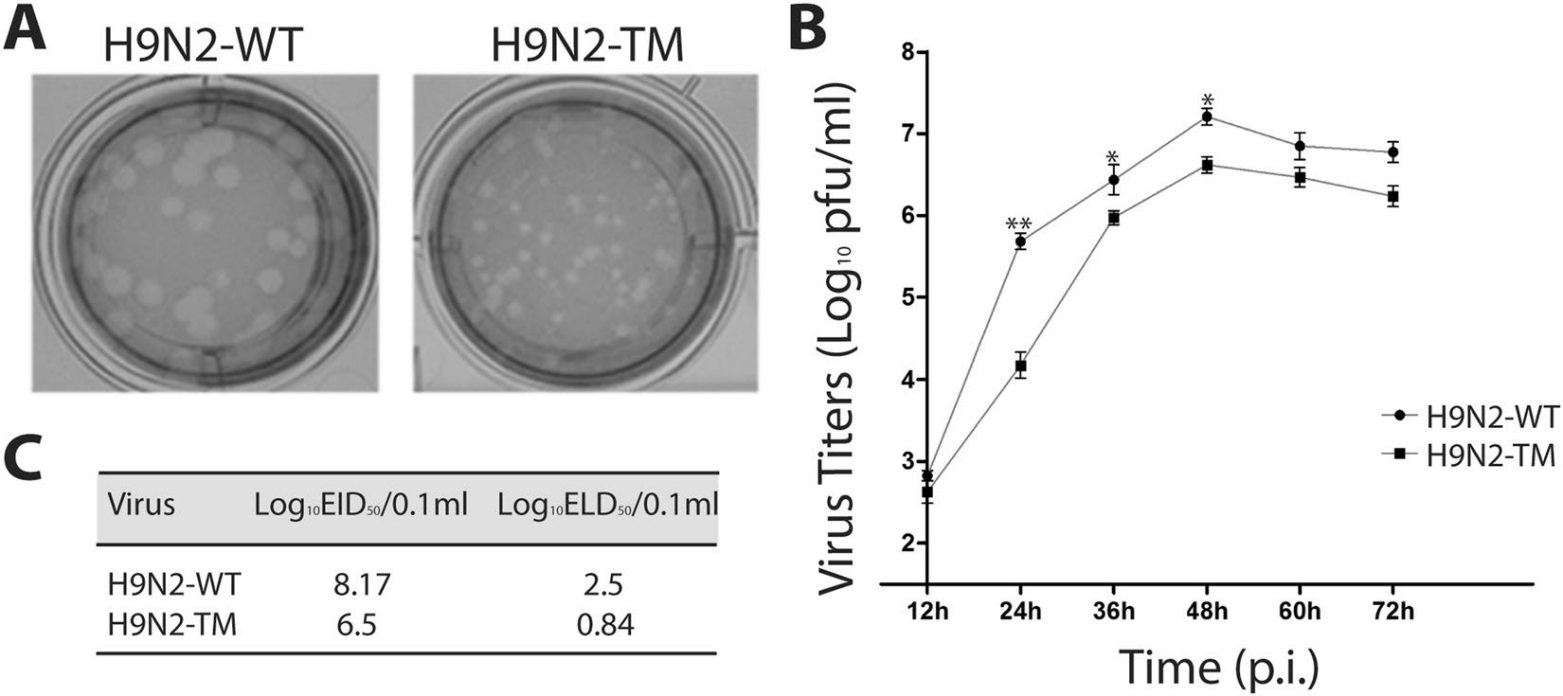
Reduced viral growth rate of TM-replaced viruses. Plaque phenotypes of H9N2-WT and H9N2-TM. (**B**) Virus growth curve of H9N2-WT and H9N2-TM recombinant viruses at different time points. Error bars represent the standard deviation from triplicate experiments. *Indicates *p* < 0.05; **indicates *p* < 0.01. (**C**) Growth properties and virulence of H9N2-WT and H9N2-TM viruses *in vivo*.

To further investigate whether the biological characteristics of the recombinant viruses were changed through the transmembrane (TM) domain replacement, MDCK cells were infected with the two recombinant viruses. We found the TM-replaced virus (H9N2-TM) formed smaller plaques than the recombinant wildtype virus (H9N2-WT) (Fig. 2A), suggesting a slow viral replication due to the transmembrane (TM) domain replacement in cells. This was further confirmed when growth curves was measured that the pfu titers of recombinant H9N2-TM were lower than that of H9N2-WT (Fig. 2B). The pfu titers of H9N2-TM were significantly decreased at 24 h (*p* < 0.01), 36 h (*p* < 0.05) and 48 h (*p* < 0.05) post-infection, suggesting TM-replacement may have an effect on delaying virus replication. When inoculated in chicken embryos, the H9N2-WT showed 8.17 log_10_EID_50_/0.1 ml for virus titer, while the recombinant H9N2-TM showed 6.5 log_10_EID_50_/0.1 ml (Fig. 2C), indicating a low infectivity of H9N2-TM in chicken embryos. Furthermore, our data suggest that the delay of the virus replication may affect virulence of the virus in chicken embryos.

To summarize, the replacement of H3 HA TM domain impeded recombinant H9N2 virus growth in MDCK cells. Moreover, the TM-replaced recombinant virus showed a reduced infectivity when inoculated in chicken embryos.

### TM-replaced viruses reduced fusion ability and increased thermal and acidic resistances

**Figure 3:**
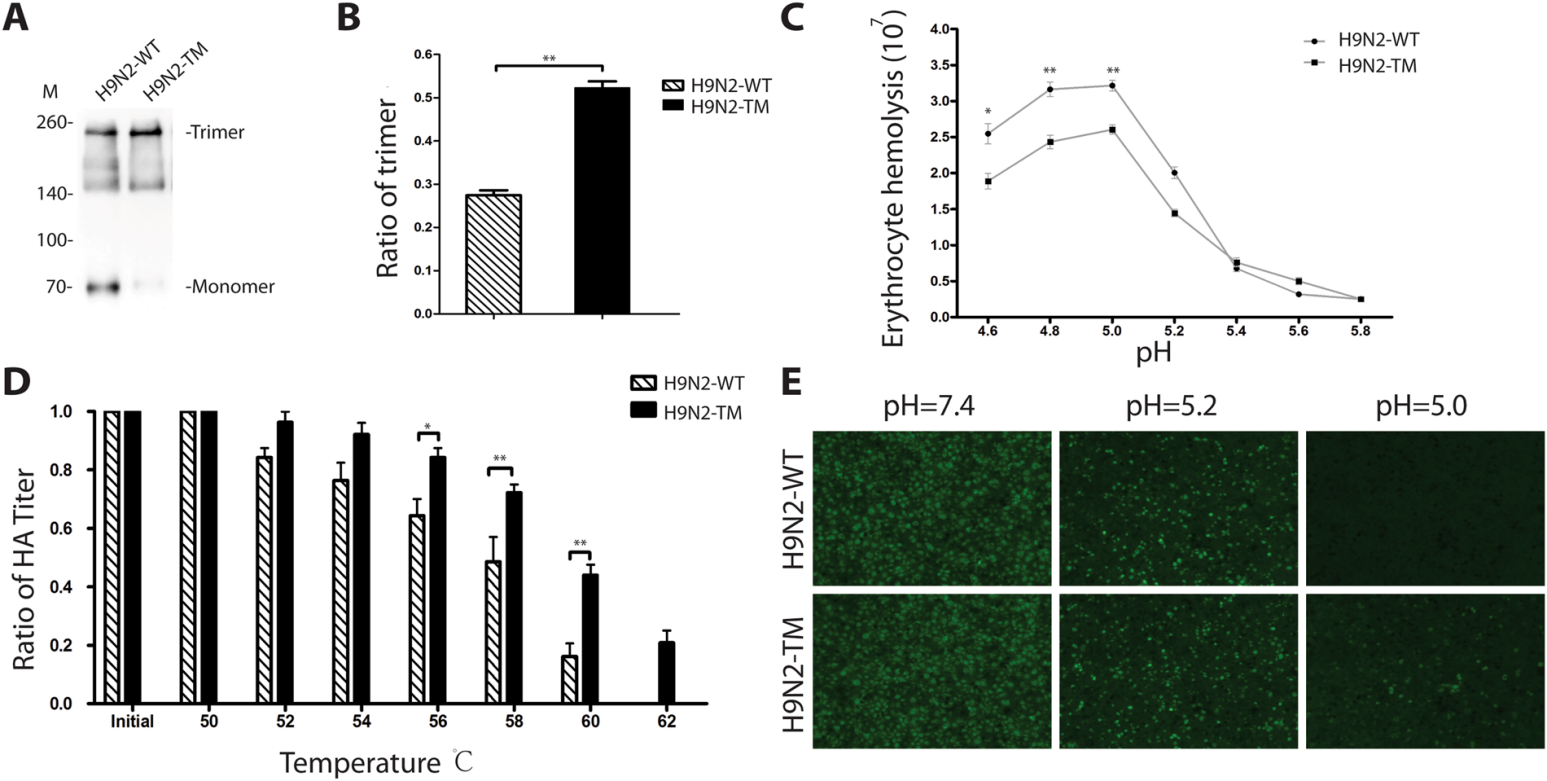
Analysis of HA trimer content, thermal and acidic resistances of the TM-replaced recombinant H9N2-TM virus. Western-blot of H9N2-WT and H9N2-TM under non-reducing conditions. (**B**) Quantification of HA trimer content in H9N2-WT and H9N2-TM. **Indicates *p* < 0.01. (**C**) Fusion activity in erythrocytes of H9N2-TM and H9N2-WT at different pH values. *Indicates *p* < 0.05; **indicates *p* < 0.01. (**D**) Thermal resistance of H9N2-WT and H9N2-TM at a temperature ranging from 50 to 62 °C. Error bars represent the standard deviation from triplicate experiments. *Indicates *p* < 0.05; **indicates *p* < 0.01. (**E)** Acidic resistance of H9N2-WT and H9N2-TM in environments of pH 5.0, 5.2, and 7.4 at 37 °C using indirect immunofluorescene assay (IFA).

In influenza viruses, the stalk domain (HA2) is crucial for the stabilization of the HA trimer, in which its transmembrane (TM) domain plays an important role in virus anchor. To further investigate whether TM-replacement may affect the stability of the HA trimer, we analyzed content of covalent bond-linked trimers of the HA protein through non-reducing western blot. The result showed that the recombinant H9N2-TM had an increased trimeric composition of the HA under non-reducing condition, comparing to H9N2-WT (Fig. 3A,B). Full-length blot is presented in Supplementary Figure 1B.

The trimeric HA is important for receptor binding and membrane fusion activity. To analyze whether the fusion activity of recombinant viruses was altered, we utilized virus-induced erythrocyte hemolysis assay. At low pH (5–6), the trimeric HA undergoes conformational changes that trigger membrane fusion. There was no difference in fusion ability at pH5.2 to pH5.8 between the H9N2-WT and H9N2-TM recombinant viruses. However, H9N2-TM showed poorer fusion activity than that of H9N2-WT in the pH ranging from 4.6 to 5.2 (P < 0.05) (Fig. 3C). This data suggests that the TM domain plays an important role in restricting the virus-induced membrane fusion activity in acidic environment.

To further investigate whether the alteration of fusion ability is due to a change in viral biological characteristics such as thermal or acidic resistances, we incubated the recombinant viruses at different temperature from 50 °C to 62 °C. The recombinant viruses H9N2-WT and H9N2-TM had similar thermal resistance under 56 °C. And their HA titers decreased gradually while the temperatures increased. From 56 °C, the HA titers of the recombinant H9N2-WT declined more than the H9N2-TM and the difference was significant (*P* < 0.05) (Fig. 3D). This data indicates that the recombinant H9N2-TM virus had an increased thermal resistance.

We next examined the infectivity of the recombinant viruses in MDCK cells in acid environment (pH 5.0, 5.2, 7.4). Fluorescent labeled viruses could be detected in each sample at pH 7.4 (Fig. 3E), suggesting that the infectivity of recombinant viruses remained normal at a neutral environment. The fluorescence receded rapidly at pH 5.2 in both groups. At pH 5.0, the fluorescence of the recombinant H9N2-WT could be barely detected, while the fluorescence of H9N2-TM could be still detected though the signal was weak. These data suggest that the recombinant H9N2-TM remains acid resistance at low pH (5.0), thus is still infectious under acid environment.

Taken together, the increased thermal and acidic resistances of the recombinant H9N2-TM virus suggest that the substitution in the transmembrane (TM) domain can affect the stability of the HA, therefore alters viral biological characteristics.

### Recombinant H9N2-TM elicited higher antibodies and provided cross-protection in mice

**Figure 4:**
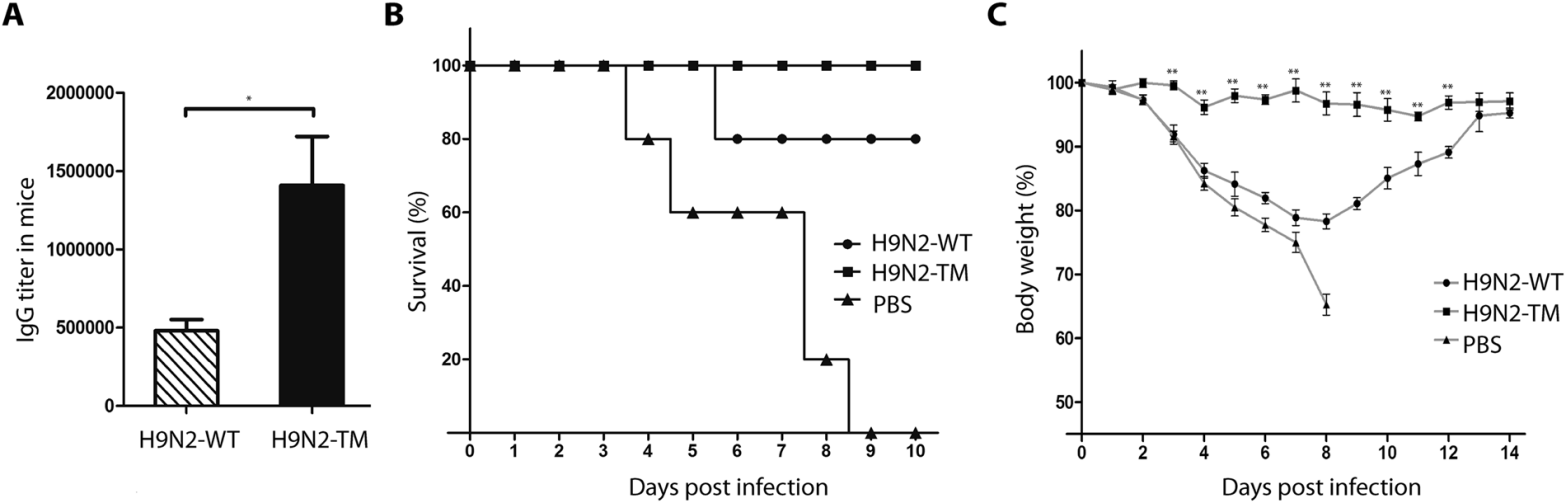
Mice immunized with H7N9-TM showed higher survival rate and less body weight loss. (**A**) Anti-HA serum IgG titers against H9N2-WT and H9N2-TM. (**B**) Survival rates of immunized mice challenged with heterologous H9N2-YYS01 strain. *Indicates *p* < 0.05; **indicates *p* < 0.01. n = 5. (**C**) Body weight loss of immunized mice challenged with heterologous H9N2-YYS01 strain. * indicates *p* < 0.05; **indicates *p* < 0.01. n = 5.

To explore whether the increased HA trimers and increased thermal and acidic resistances could result in better immune responses thus providing better protection in mice, we first vaccinated six-week-old mice with inactivated recombinant H9N2-WT and H9N2-TM viruses twice to check antibody responses. A significant increase of the serum HA-specific IgG antibody titers was observed in the H9N2-TM group (*p < *0.05) (Fig. 4A).

**Figure 5:**
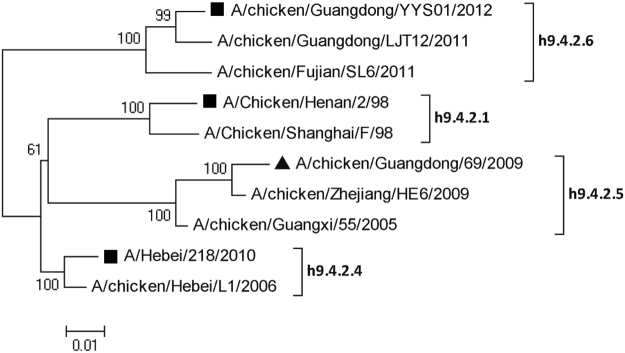
Phylogeny of HA genes of H9N2 strains of different clades. The scale bar represents a 1% nucleotide change. H9N2 strain labeled with black triangle was used for virus rescue and TM-replacement. H9N2 strains labeled with black squares were used in HI test and challenge assay.

To determine the level of protection against challenges of homologous H9N2 virus, mice were then challenged with 3x MLD_50_ of mouse-adapted wild-type A/Chicken/Guangdong/YYS01/2012 (H9N2) virus in a different clade (Fig. 5). All mice in the control group succumbed to infection by day 9 post infection, while all animals were completely protected in the H9N2-TM vaccinated group (Fig. 4B). Comparing to the H9N2-TM vaccinated group, mice immunized with H9N2-WT showed significant weight loss as the PBS group from day 3 post infection (Fig. 4C). These data indicate that TM-replacement could provide better cross-protection against inter-clade challenge in mice.

### H9N2-TM elicited higher HI titer and provided cross-protection in chickens

**Figure 6:**
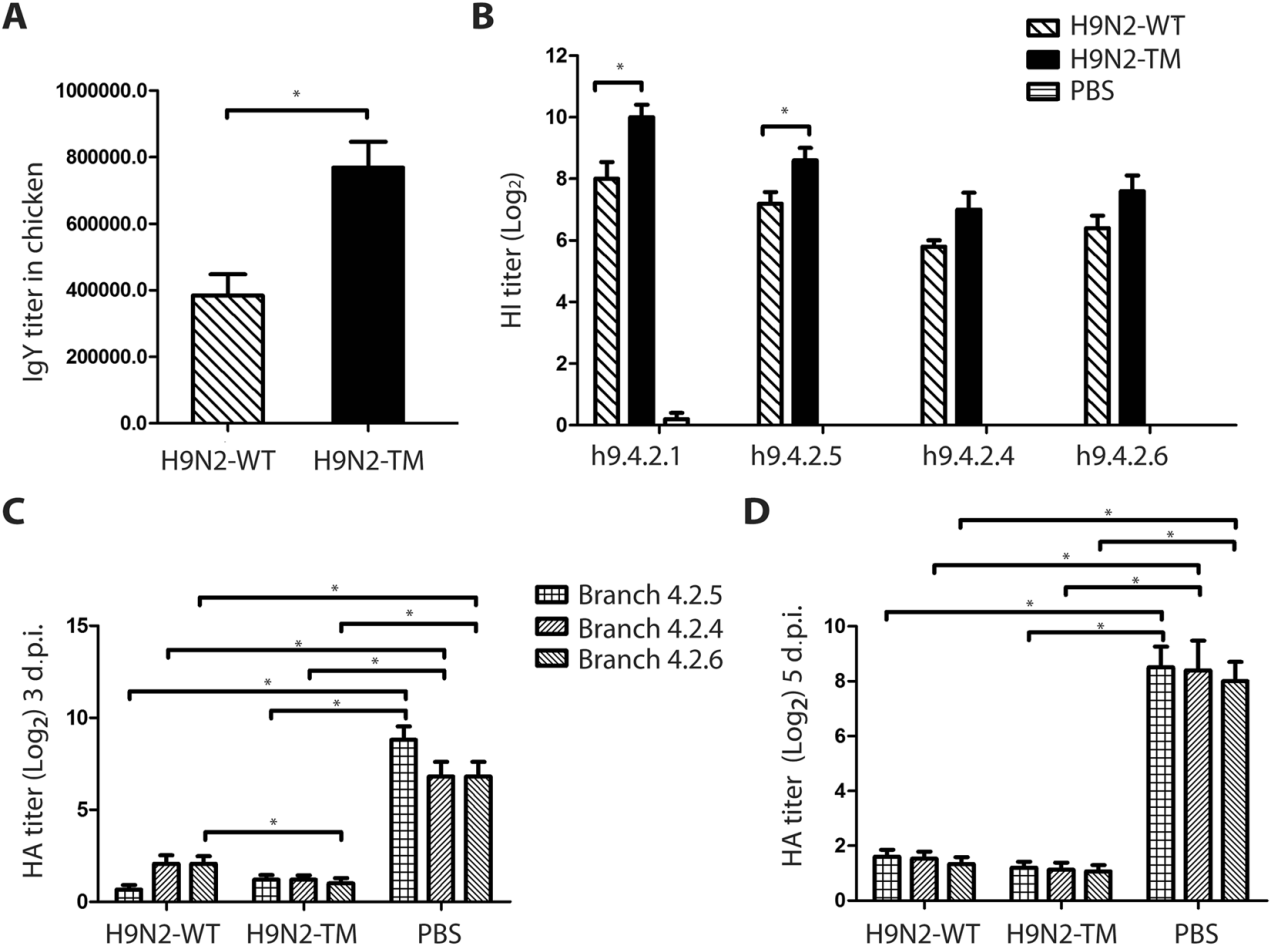
Analysis of Anti-H9 HA IgY and HI titers in SPF chicken. (**A**) Anti-HA serum IgY titers against H9N2-WT and H9N2-TM. (**B**) HI titers of H9N2 viruses of different phylogenetic H9N2 clades. (**C**) HA titers 3 d.p.i. of different clades of H9N2 (4.2.4, 4.2.5 and 4.2.6). (**D**) HA titers 5 d.p.i. of different clades of H9N2 (4.2.4, 4.2.5 and 4.2.6). Error bars represent arithmetic mean antibody titers ± standard errors. *Indicates *p* < 0.05.

Since H9N2 is an avian originated virus, we next explored whether a better immune responses could be induced by H9N2-TM in chickens. We performed ELISA assay and HI assay to analyze the antibody responses in vaccinated chickens. In ELISA, H9 HA protein was utilized as antigen. Similar as the antibody response results in mice, we found that the IgY titers of H9N2-TM vaccinated group were significantly higher, comparing to that of the H9N2-WT group (*p < *0.05) (Fig. 6A). In HI assay, inactivated H9N2 viruses of different clades (h9.4.2.1, h9.4.2.4, h9.4.2.5 and h9.4.2.6) were utilized as antigens (Fig. 5). When viruses from clades 4.2.4 and 4.2.6 were used as antigens, The HI titers of H9N2-TM vaccinated chicken sera were comparable with the H9N2-WT vaccinated group. However, when viruses from clades 4.2.1 and 4.2.5 were used as antigens, the HI titer of H9N2-TM group was significant higher than that of H9N2-WT (*p* < 0.05) (Fig. 6B).

To further investigate the cross-protection reaction through TM replacement, chickens were challenged using different phylogenetic H9N2 viruses from clade 4.2.4, 4.2.5 and 4.2.6 (Table 1) (Fig. 5). Virus shedding determined by HA titers was utilized to elucidate protection provided by vaccines. Chickens immunized with H9N2-WT and H9N2-TM showed complete protection challenge by clade 4.2.5. Virus shedding was found in all groups immunized with PBS. No virus shedding was found in all groups immunized with H9N2-TM. At day 3 post infection, the shedding rates of H9N2-WT groups was 20% (virus shedding was found in 3 out of 15 chickens) and 13.3% (virus shedding was found in 2 out of 15 chickens), which are more than the H9N2-TM vaccinated groups, when challenged with viruses from branch 4.2.6 or branch 4.2.4 respectively. Furthermore, the HA titers of virus shedding in H9N2-WT vaccinated group was significantly higher (*p* < 0.05) than that of the H9N2-TM group, at 3 d.p.i when challenged with viruses from clade 4.2.6 (Fig. 6C). The HA titers of 5 d.p.i were comparable in all H9N2-WT and H9N2-TM vaccinated groups (Fig. 6D). The differences in virus shedding suggest a better inter-clade cross-protection when the recombinant H9N2-TM virus was used as vaccine.

**Table 1:** Virus shedding after challenge with different H9N2 viruses.

Strains for challenge	Recombinant virus for immunity	Virus shedding	
		Day3 p.i.	Day5 p.i.
H9N2-Branch4.2.5	H9N2-WT	0/15	0/15
	H9N2-TM	0/15	0/15
	PBS	5/5	5/5
H9N2-Branch4.2.4	H9N2-WT	3/15	0/15
	H9N2-TM	0/15	0/15
	PBS	5/5	5/5
H9N2-Branch4.2.6	H9N2-WT	2/15	0/15
	H9N2-TM	0/15	0/15
	PBS	5/5	5/5

In conclusion, our data suggest that the recombinant TM-replaced H9N2-TM recombinant virus can provide better inter-clade cross-protection than the wild type.

## Discussion

One characteristic of influenza A virus is that it undergoes rapid antigenic variation, especially under immunological pressure. Mutations on hemagglutinin (HA) and neuraminidase (NA) usually cause antigenic drift and antigenic shift, thus resulting in virus immune escape. Therefore, it is difficult for the existing avian influenza inactivated vaccines to deal with the new strains, due to lack of cross-immune protection. Take H9N2 viruses in China for instance, which was previously considered to share similar antigenicity profiles^2,21^, vaccine strains and their relevant variants are mainly used for H9N2 virus protection in China. However, current existing vaccines could not fulfill their task to prevent the outbreaks of H9N2 in 2011–2013 in poultry farming of China^22^. Moreover, recent studies found that some H9N2 influenza viruses were originated from the vaccine strains through antigenic drift and these antigenic variants finally caused the catastrophe in poultry industry of China in 2011–2013, even under the long-term vaccination programs^9^. Therefore, to prevent next outbreaks of further antigenic H9N2 variants caused by immunological pressure, an effective, safe and subtype-specific H9N2 vaccine which can provide cross-protection is required.

The HA stem has recently drawn attention to be a new potential influenza candidate site for universal vaccine development. Since the discovery of a series of broadly neutralizing monoclonal antibodies, the majority of which target to highly conserved region of HA stem, researchers began to discuss the potential to induce broad protective immunity^23–26^. Various attempts were conducted to stabilize the HA stem in order to induce broad protective immunity. The stability and immunogenicity of the stabilized HA proteins through fusion with trimerization sequences have shown increased broad protective immunity. For instance, researchers showed when the HA protein was fused to the foldon domain of fibritin from bacterial phage T4 or GCN4p trimerization repeat, the fusion proteins showed increased stability and cross-reactive immunity^27,28^. Further researchers showed that fused ectodomain of HA protein to ferritin could form nanoparticles and the nanoparticle vaccine could improve the potency and breadth of influenza virus immunity^29^, suggesting the importance of HA protein stability in enhancing cross-protection. This plausible correlation of the major surface antigen stability and cross-immunity was also supported by other researches using S protein of SARS virus that the trimerized forms could elicit higher levels of neutralizing antibodies^30^. The biological significance of HA stability remains unclear and future work is required.

Our previous studies found that the transmembrane domain of H3 subtype HA protein had a unique microdomain, which was related to the stability and cross-immunity of influenza virus^19,31–33^. Furthermore, we found that H3-WT TM-dependent cross-protection could be transferred to other subtypes by replacing their TMs with H3-WT TM^19^, suggesting a plausible correlation of HA stability and cross-immunity. Recently, we developed a TM-replaced H7N9 vaccine. Mice vaccinated with H7N9-TM vaccine strain showed increased cross-reactive antibodies and were well protected against interclade H7N9 viruses^20^. Consistent with the previous studies, our data indicated that TM replacement could actually alter the physical and chemical characteristics of H9N2 virus. The delayed virus replication in TM-replaced viruses may contribute to low pathogenesis. The increased thermal and acidic resistances suggest that a modification on transmembrane (TM) domain might lead to structural change of the HA protein, thus leading to an alteration of the biological characteristics in the recombinant virus.

When used as a vaccine, the recombined TM-replaced H9N2 strain (H9N2-TM) could perform better subtype-specific protection and inter-clade cross-protection against different phylogenetic H9N2 viruses compared to that of recombinant H9N2-WT in both mice and chickens, suggesting the TM-replaced H9N2 vaccines are efficient in different species. The elicited HA-specific antibody level was comparable in both H7N9^20^ and H9N2 viruses, when TM-replacement was introduced in the vaccine strains. Therefore, our results have suggested a universal technique to improve the existing influenza H9N2 inactivated virus vaccine. Furthermore, according to our researches, this technique could be used to develop vaccines of various types of avian influenza viruses. This TM-replacement technique suggests a way to improve the existing vaccine immune broad-spectrum activity, prolong the shelf life of the vaccine. And therefore, it is a good candidate for prevention of avian influenza and the development of new generation of vaccines in the future.

In conclusion, in this study, our results demonstrated that inter-clade cross-protection could be enhanced with TM replacement and therefore further suggested a plausible correlation of HA stability and cross immunity. Our results demonstrate that the TM-replaced vaccine could be a more effective influenza vaccine candidate and thus suggest a strategy to develop effective subtype-specific H9N2 vaccine or even heterosubtypic vaccines against influenza viruses. Other questions like whether the effect of TM-replacement is H3 specific, which segment is responsible for transmembrane domain function, whether TM-replacement changes the virus structure and how TM-replacement influences virus’s biological characteristics still remain unknown. And future work is required to answer these questions.

## Materials and Methods

### Cells and viruses

Human embryonic kidney cells (293 T) and Madin-Darby Canine Kidney (MDCK) cells were cultured in Dulbecco’s modified Eagle’s medium (DMEM) supplemented with 10% fetal bovine serum (FBS) (Thermo Scientific), penicillin (100 units/ml), streptomycin (100 μg/ml) in an atmosphere of 5% CO2 at 37 °C. H9N2 strains A/Chicken/Guangdong/69/2009 (H9N2) (GenBank accession no. KF514116.1), A/Chicken/Guangdong/YYS01/2012 (H9N2) (GenBank accession no. KJ768995.1), A/Chicken/Henan/2/98 (H9N2) (GenBank accession no. AF461517.1) and A/ Hebei/218/2010 (H9) (GenBank accession no. KC296446.1) were isolated in Guangdong Province, China. The handling of experiments with live viruses was conducted in a biosafety 2 plus facility under the guidelines issued by China authority.

### Virus rescue and reverse genetics

The plasmid pHW2000 was used for reserve genetics as described previously^34^. To construct recombinant H9N2 virus containing a TM from H3 HA, strain A/Chicken/Guangdong/69/2009 (H9N2) (GenBank accession no. KF514116.1) was selected and the TM domain was replaced with a TM from H3 HA using overlap PCR (Fig. 1A). For virus rescue, 293T cells were transfected with eight genome-sense plasmids using X-tremeGENE 9 DNA transfection reagent (Roche) according to manufacturer’s instruction. Thirty six hours after transfection, TPCK-treated trypsin (Sigma) was added to the cells with a final concentration of 0.5–1 μg/ml. The transfected cell culture supernatant was collected at 48–60 h post-transfection and used to passage onto 10-day-old SPF embryonated chicken eggs for the propagation of the recombinant viruses. The rescued recombinant virus containing WT HA was designated as H9N2-WT, whereas the rescued virus containing H3 HA was designated as H9N2-TM.

### Virus propagation and purification

Both recombinant H9N2-WT and H9N2-TM viruses were propagated in 10-day-old SPF embryonated chicken eggs. After 72 h, the allantoic fluids were collected and inactivated with 0.1% β-propiolactone (BPL) at 4 °C for 24 h. The inactivated viruses were tested by performing serial passages on SPF embryonated chicken eggs. The purification was performed by centrifugation on 20–50% sucrose density gradients as described previously^35^.

### Western blot and electron microscopy

DS-polyacrylamide gel electrophoresis (SDS-PAGE) was performed to validate the purified virions. Non-reducing western blot was performed as previously described^32^. Anti-H9N2 mouse serum (1:3000) was used as a primary antibody. The negative staining of purified recombinant virions was done as described previously^36^. Briefly, virions were stained by the phosphotungstic acid buffer and the shape was photographed on JEM-100 CX-II electron microscope (JEOL).

### Virus growth and plaque assay

MDCK cells were cultured in 24-well plates and inoculated with viruses (m.o.i. = 0.001) for 1 h. Supernatants were collected every 12 h until 72 h post-infection. The viral titers in the supernatants were determined by plaque assay in MDCK cells. Briefly, MDCK cells in 12-well plates were infected with serial tenfold dilutions of the recovered viruses for 1 h at 37 °C. Then the infected cells were washed three times with PBS and incubated at 37 °C for 3 days with MEM-2% agarose medium containing 2 μg/ml of trypsin (Sigma). Cells were stained with neutral red and the formed plaques were photographed.

### Thermal and acidic resistance assays

The resistance assay was performed as described before^31^. Briefly, the viruses with the same HA titer were incubated at a temperature ranging from 50 to 62 °C for 20 min. The HA titres were measured subsequently when viruses were cooled down to room temperature. In the acidic resistance assay, the viruses with the same pfu were incubated in an acidic buffer (10 mM HEPES, 10 mM MES in PBS) at pH 7.4, 5.2, and 5.0 at 37 °C for 30 min. The solutions were then adjusted to pH 7.0. MDCK cells were infected with the recombinant viruses in 24-well plates at an m.o.i. of 2 for 30 min at 37 °C, then the media were replaced with serum-free media containing 2 μg/ml TPCK-trypsin. The infected plates were fixed with 4% paraformaldehyde, permeabilized with 0.2% Troton X-100 in PBS, and stained with the FITC-labeled mAb against NP (Abcam). The cell images were taken under inverted fluorescence microscope (Zeiss).

### Virus-cell fusion assay

According to standard protocol described previously^37^, viruses standardized to 256 HA units were mixed with 2% chicken red blood cells (RBC). The pH was adjusted from 5.8 to 4.6 with addition of the sodium citrate buffer. After 30 min incubation, supernatants were transferred to an ELISA plate for determination of NADPH content by optical density measurement (340 nm) with a Bio-Tek ELISA plate reader (Bio-Tek Instruments). Baseline NADPH activity values were derived from samples without viruses that underwent identical treatment.

### Animal challenge

15-day-old SPF chickens were housed in individual isolators under positive pressure and randomly divided into three groups. Each group (n = 15) was subcutaneously immunized together with mineral oil adjuvant once. Blood samples were then collected two weeks after immunization. Three weeks after immunization, vaccinated chickens were challenged intranasally with a dose (100 × EID50) of the following H9N2 viruses, A/Chicken/Guangdong/YYS01/2012 (H9N2) (GenBank accession no. KJ768995.1), A/Chicken/Henan/2/98 (H9N2) (GenBank accession no. AF461517.1) and A/ Hebei/218/2010 (H9) (GenBank accession no. KC296446.1). Swabs of the larynx and cloaca were collected at 3 and 5 days post infection to inoculate SPF chicken embryos subsequently. Allantoic fluid were collected after 72 hours incubation at 37 °C to determine HA titres. Samples were regarded as expelling viruses when titers ≥ 2^4^.

Six-week-old female BALB/c mice were selected and intramuscularly immunized by inactivated H9N2-TM and H9N2-WT viruses, with Freund’s incomplete adjuvant respectively, twice on week 0 and 2. Blood samples were collected at week 4.Three weeks after booster immunization, mice were challenged intranasally with mouse adapted A/Chicken/Guangdong/YYS01/2012 (H9N2) (3 × MLD50). Survival rate and weight loss were monitored daily after the challenge.

### ELISA for anti-HA IgG antibodies

HA-specific anti-chicken immunoglobulin Y (IgG) isotype antibody titers in chicken sera and IgG antibody titers in mice sera were determined using enzyme-linked immunosorbent assay (ELISA). H9 HA protein with a concentration of 3 μg/ml were coated, incubated with serial dilutions of each serum sample(37 °C for 1 h) and detected by HRP-conjugated goat anti-chicken IgG (Proteintech) and HRP-conjugated goat anti-mouse IgG antibodies (Proteintech). Optical densities were read at 450 nm using a spectrophotometer (Bio-Tek).

### Hemagglutination inhibition assay

25 μl of each influenza virus of four HA units was used in the HI assay. Each sample was treated with receptor destroying enzyme (RDE) and diluted in a 2-fold serial. The highest dilution of the serum able to inhibit hemagglutination was defined as the HI titer.

### Ethics statement

The virus propagation studies in embryonated eggs were approved by the Institutional Animal Care and Use Committee of Sun Yat-sen University. Animal experiments were approved by the Institutional Animal Care and Use Committee of Sun Yat-sen University and performed in accordance with the guidelines of the Sun Yat-sen University Institutional Animal Care and Use Committee. Research was conducted in the compliance with guidelines of the Ordinance on Laboratory Animals Management set by the State Scientific and Technological Commission of China.

### Statistical analysis

Data were presented as mean ± SEM from at least three independent experiments. Unless otherwise noted, statistical analyses were performed using Student’s two-tailed t test. Difference was considered statistically significant at P < 0.05.

### Data Availability

The datasets generated during and/or analyzed during the current study are available from the corresponding author on reasonable request.

## Conclusion

Taken together, our data indicate that H3 TM domain is critical for biological characteristics and inter-clade cross-protection of influenza viruses, and subtype-specific H9N2 cross-protection can be enhanced with H3-TM replacement. Our results suggest a plausible correlation of HA stability and immunity. Therefore, TM-replaced vaccine can be a more broad-spectrum influenza vaccine candidate.

## Electronic supplementary material

Supplemental Figure 1
