# Supplementary material for: Recombinant influenza H9N2 virus with a substitution of H3 hemagglutinin transmembrane domain showed enhanced immunogenicity in mice and chicken
Source: Sci Rep. 2017 Dec 20;7:17923. doi: 10.1038/s41598-017-18054-x (PMC5738434; doi:10.1038/s41598-017-18054-x)
Supplement: Supplementary file 1 — Supplemental Figure 1 [file 41598_2017_18054_MOESM1_ESM.pdf]

Recombinant influenza H9N2 virus with a substitution of H3 hemagglutinin transmembrane domain showed enhanced immunogenicity in mice and chicken

Yun Zhang, Ying Wei, Kang Liu, Mengjiao Huang, Ran Li, Yang Wang, Qiliang Liu, Jing Zheng, Chunyi Xue, Yongchang Cao*

State Key Laboratory of Biocontrol, College of Life Sciences, Sun Yat-sen University, Guangzhou, 510006, People's Republic China

*Correspondence: caoych@mail.sysu.edu.cn

Yongchang Cao, PhD, Professor

Address: Life Sciences School, Sun Yat-sen University, Guangzhou Higher Education Mega Center, P.R.China (510006)

Phone: 86-020-39332934

Fax: 86-020-39332841

E-mail: caoych@mail.sysu.edu.cn

A

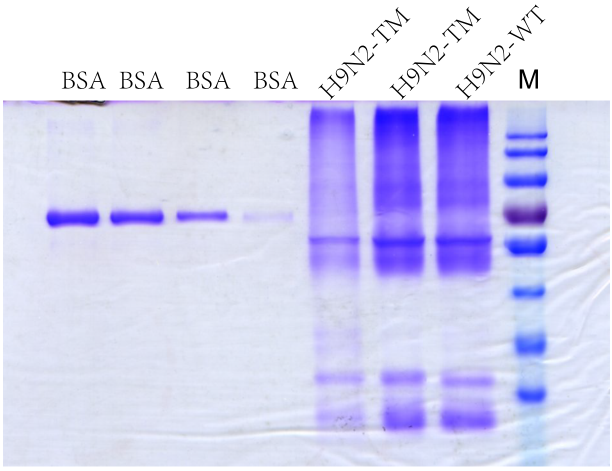
BSA
BSA
BSA
BSA
H9N2-TM
H9N2-TM
H9N2-WT
M

B

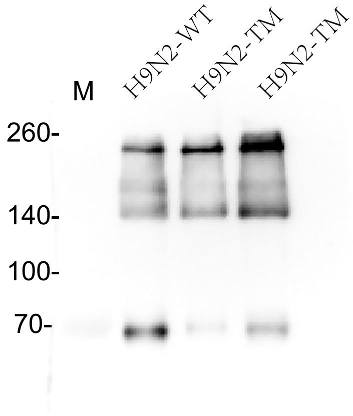
M
H9N2-WT
H9N2-TM
H9N2-TM
260-
140-
100-
70-

Supplemental Figure 1. Full length blots of Figure 1C and Figure 3A.

(A) Full length blot of Figure 1C. (B) Full length blot of Figure 3A.

.
